# Lung Cancer Proteomics: Recent Advances in Biomarker Discovery

**DOI:** 10.1155/2011/726869

**Published:** 2011-09-15

**Authors:** Paola Indovina, Eleonora Marcelli, Pasquale Maranta, Giulio Tarro

**Affiliations:** ^1^Department of Human Pathology and Oncology, University of Siena, 53100 Siena, Italy; ^2^Sbarro Institute for Cancer Research and Molecular Medicine and Center for Biotechnology, College of Science and Technology, Temple University, Philadelphia, PA 19122, USA; ^3^Terni Human Health Bioscience Institute, Human Health Foundation, 05100 Terni, Italy; ^4^Unit of Virology, “D. Cotugno” Hospital, 80123 Naples, Italy; ^5^Committee on Biotechnologies and VirusSphere, World Academy of Biomedical Technologies, UNESCO, 75732 Paris, France

## Abstract

Lung cancer is the most common cause of cancer death in both men and women in Western countries, with a 5-year survival rate of 15%, which is among the lowest of all cancers. The high mortality from lung cancer is due not only to the late stage diagnosis but also to the lack of effective treatments even for patients diagnosed with stage I lung cancer. Therefore, there is an urgent need to identify new markers for early diagnosis and prognosis that could serve to open novel therapeutic avenues. Proteomics can represent an important tool for the identification of biomarkers and therapeutic targets for lung cancer since DNA-based biomarkers did not prove to have adequate sensitivity, specificity, and reproducibility. In this paper we will describe studies focused on the identification of new diagnostic, prognostic, and predictive markers for lung cancer, using proteomics technologies.

## 1. Introduction

Lung cancer is the most common cause of cancer death in both men and women in Western countries, accounting for 30% of cancer-related mortality in the United States every year [1]. The number of deaths from lung cancer is about three times higher than that from prostate cancer among men and about twice that from breast cancer among women. The most important risk factor for the development of lung cancer is smoking, with a risk in smokers on average tenfold higher than in nonsmokers. Lung cancer is generally divided into small-cell lung cancer (SCLC), representing approximately 15% of cases, and non-small-cell lung cancer (NSCLC), representing 85% of cases and including several histological types, such as adenocarcinoma, large-cell carcinoma, and squamous-cell carcinoma [2]. Regardless of subtype, the 5-year survival rate for lung cancer is among the lowest of all cancers (approximately 15%) [1, 3]. SCLC is highly responsive to chemotherapy and radiation therapy but it is often widely disseminated by the time of diagnosis, rendering the cure difficult. In contrast to SCLC, NSCLC shows a strong primary resistance to anticancer drugs. At diagnosis, patients with NSCLC can be divided into three groups based on the extent of the disease and the therapeutic strategy used: the first group of patients, accounting for approximately 30% of cases, is diagnosed at an early disease stage and has tumors that are surgically resectable; the second group (20% of cases) includes patients with either locally and/or regionally advanced tumors that are treated with a combination of chemotherapy and radiotherapy; finally, the third group (half of patients) comprises patients with distant metastasis at the time of diagnosis. For this group the only treatments available are chemotherapy or radiation therapy for palliation of symptoms. Thus, it is evident that lung cancer is a heterogeneous disease both for its biological features and for its clinical management [2]. The high mortality from lung cancer is due not only to the late stage diagnosis, when cure is very unlikely, but also to the lack of effective treatments even for patients diagnosed with stage I lung cancer, whose survival is also surprisingly low [1]. Therefore there is a great need to identify new markers for early diagnosis and prognosis that could open the way for the development of new therapeutic strategies. A number of potential biomarkers have been identified, such as mutations in *KRAS* and *TP53* and alterations in expression of carcinoembryonic antigen (CEA), cytokeratin-19 fragment (CYFRA21-1), neuron-specific enolase (NSE), and cancer antigen-125 (CA-125). However, few have proved to be useful in the clinic, showing low sensitivity, specificity, and reproducibility [4, 5].

Previously, we identified a ~100 kDa protein, which is part of a protein complex named tumor liberated proteins (TLP), as a promising blood marker for early diagnosis of lung cancer [5, 6]. In particular, this protein proved to have high specificity and sensitivity for stage I patients with NSCL. TLP might also represent a predictive marker of cell transformation since it is expressed in interstitial lung fibrosis. Moreover, TLP showed a specific immunogenic activity, suggesting its possible use as an anticancer vaccine. Indeed, it is able to induce delayed hypersensitivity reactions and to promote blastogenesis in cultured lymphocytes from patients presensitized with TLP. Research is ongoing to obtain the complete sequence of TLP, by proteomics approaches, in order to achieve adequate antigen preparations that might be used to generate assays for early diagnosis and, possibly, a specific anticancer vaccine. 

Proteomics is becoming an increasingly important tool for the identification of biomarkers and therapeutic targets for cancer. The standard proteomics techniques, namely, two-dimensional gel electrophoresis (2DE) and mass spectrometry (MS), have been developing over the past three decades, but only at the end of 90s, through the development of high-throughput platforms, proteomics was no longer limited to the analysis of a few proteins at a time but allowed the simultaneous measurement of multiple protein products and/or protein modifications (for a detailed discussion of these methods refer to other publications [7, 8]). Therefore, it is now possible to detect crucial molecular patterns in malignant cells, which might indicate disease progression or response to therapy. Moreover, proteomics can represent an advancement over genomics because protein biomarkers can be a more accurate signature of a disease state since proteins and not transcripts are the actual functional players [9]. Indeed, mRNA levels not always reflect protein expression or activity, due to a number of posttranslational modifications such as ubiquitination, protease cleavage, glycosylation, phosphorylation, methylation, and acetylation [4]. Therefore, it is increasingly evident that proteome investigations can lead to the identification of more reliable cancer biomarkers. For these reasons, proteomics analysis can be particularly useful to identify new biomarkers for lung cancer, for which DNA-based biomarkers did not prove to have adequate sensitivity, specificity, and reproducibility [4]. 

In this paper we will describe studies focused on the identification of new diagnostic, prognostic, and predictive markers for lung cancer, using proteomics technologies. We will discuss the most promising findings, which could be useful to improve the management of this disease.

## 2. Tissue Types for Biomarker Detection

Various tissues can be used as a source of proteins for lung cancer proteomics analyses, including cancer tissue, blood, and pleural effusions. 

Surgical specimens are principally derived from NSCLC because, as stated above, SCLC is often diagnosed when the disease has already spread and therefore surgical samples are rarely obtained [2]. Another limit is the fact that adjacent to islands of tumor cells there are stromal components, inflammatory infiltrations, and necrotic areas. Therefore, in order to limit the confounding effect of these other tissues, it is necessary to use methods, such as laser capture microdissection of tissue samples on microscope slides, for isolating only cancer cells. 

Blood proteomics analysis could have a great advantage over proteomics conducted in lung cancer tissues because blood samples are more readily accessible. Blood contains both potential biomarkers found in biopsied cancer and many circulating proteins generated in the diseased tissue [4]. However, detection of low-abundance tumor proteins in the complex and dynamic mixture of plasma proteins can be very difficult, and it is often necessary to deplete abundant serum proteins in order to reduce this complexity. Moreover, before searching biomarker proteins in blood, it can be necessary to separate proteins by their characteristics, such as glycosylation or phosphorylation, ion charges, hydrophobicity or hydrophilicity, and molecular weights by chromatographic methods. 

Although blood is the biological fluid traditionally used in biomarker studies, pleural effusions might be a new source of more specific lung cancer markers [2]. Pleural effusion protein composition is very similar to plasma but the vicinity of this fluid to tumor cells suggests an enrichment of tumor-derived proteins.

## 3. Diagnostic Biomarkers

Since cancer is characterized by a chronic active inflammation state, its microenvironment frequently contains infiltrated inflammatory cells and proinflammatory cytokines. In response to inflammation, acute-phase reactant proteins (APRPs) are produced. The association between APRP altered levels and cancer has long been established but, only recently, proteomics studies showed that APRP alterations are different in distinct tumor types [10]. Therefore, APRPs can be used as potential biomarkers for the diagnosis of different types of cancer. Among APRPs, the Haptoglobin (Hp) *β* chain [11], serum amyloid A (SAA) [12], and apolipoprotein A-1 (Apo A-1) [13] proteins represent novel potential diagnostic markers for lung cancer.

Hp is a tetrameric (*α*2*β*2) glycoprotein mainly synthesized in liver during inflammation and infection. An increase in Hp levels has also been reported in several cancers, such as breast cancer [14], ovarian cancer [15], pancreatic cancer [16], malignant lymphoma [17], urogenital tumor [18], and bladder cancer [19]. The main function of Hp is to remove free plasma hemoglobin [20] but Hp is also involved in angiogenesis [21] and cell migration [22]. In a recent study, serum level of Hp has been compared in patients with lung cancer, other types of solid cancers, and respiratory diseases and healthy donors by liquid chromatography-electrospray ionization-tandem mass spectrometry (LC-ESI-MS/MS), Western blotting, and ELISA [11]. A higher level of Hp was present in the sera of lung cancer patients with respect to healthy controls but only the Hp *β* chain showed a significant difference between lung cancer and other tumors. Therefore, the Hp *β* chain seems to be a more specific diagnostic marker for lung cancer. However, caution is needed when the Hp *β* chain is to be used as a marker to differentiate lung cancer from other respiratory diseases because Hp *β* chain levels overlap between these pathologic states. 

SAA proteins are a family of apolipoproteins with several roles, including the transport of cholesterol to the liver, the recruitment of immune cells to inflammatory sites, and the induction of enzymes degrading extracellular matrix [23]. Among the members of this family, SAA1 and SAA2 are synthesized in response to cytokines released by activated monocytes/macrophages. These proteins are produced predominantly by the liver but they were found at elevated levels in several cancers [24]. Recently, SAA1 and SAA2 were proposed to be specific diagnostic markers for lung cancer since they are expressed at higher levels in blood and cancer tissues from patients with lung cancer compared to samples from healthy donors and patients with other types of cancer or respiratory diseases, as demonstrated by LC-MS/MS, ELISA, and immunohistochemistry analyses [12]. Moreover, SAA1 and SAA2 seem to be also involved in lung cancer metastasis, by inducing the expression of matrix metallopeptidase-9 (MMP*-*9) by macrophages. Therefore, SAA1 and SAA2 could also represent new potential therapeutic targets for the inhibition of lung cancer metastasis. 

Consistent with the upregulation of SAA1 and SAA2 in lung cancer, a decreased level of Apo A-1, an APRP responsible for endogenous cholesterol removal from tissues, was observed in sera of adenocarcinoma patients with respect to healthy donors [13]. In fact, following acute-phase reaction, Apo A-1 is replaced by SAA, which becomes the predominant apolipoprotein implicated in the removal of cholesterol at inflammatory sites [25]. Therefore, together with the increase in SAA1 and SAA2, the decrease in Apo A-1 could also be considered a potential lung cancer marker.

As stated above, due to their vicinity to tumor cells, pleural effusions could be enriched of lung cancer-related proteins and therefore can be a useful source of biomarkers [2]. In a recent study, the proteome of serum and pleural effusions was compared between NSCLC patients and benign lung diseases using two-dimensional difference gel electrophoresis (2D-DIGE). As expected, more potential cancer biomarkers were found in pleural effusions than in serum [26]. Among the candidate markers, the most interesting were gelsolin, a protein possibly involved in cancer invasion, the metalloproteinase inhibitor 2, implicated in lung parenchyma disorganization, and the pigment epithelium-derived factor (PEDF), involved in angiogenesis inhibition. Another potential diagnostic marker found in pleural effusions is NPC2 (Niemann-Pick disease type C2 protein), a protein that seems to be involved in regulating the transport of cholesterol [27]. Although it is not clear whether NPC2 could play a role in tumor development, it was found to be upregulated in patients with adenocarcinoma compared with inflammatory pleuritis by isoelectric focusing- (IEF-) LC-MS/MS. 

Glycosilated proteins could be a potential source of new biomarkers because they represent 50% of the secreted proteome and serum proteins in cancer patients are known to be further glycosylated. Therefore, glycoproteome analysis could have great advantages for cancer biomarker discovery [4, 28]. Glycoproteomics studies, performed by different methods for glycoprotein fractionation (multilectin chromatography or N-GP capture) followed by LC-MS/MS, revealed potential lung cancer biomarkers, such as plasma kallikrein (KLKB1) [29], pleural effusion periostin, multimerin-2, CD166, and lysosome-associated membrane glycoprotein-2 (LAMP-2) [30]. 

It has been suggested that the diagnosis based on the measurement of a panel of biomarkers could be more reliable than a single marker test [31]. Consistently, Patz and colleagues demonstrated that four markers (retinol binding proteins and 1-antitrypsin, discovered by proteomics, and CEA and squamous cell carcinoma antigen, previously known to be cancer associated) have inadequate diagnostic power when tested independently but proved clinical utility when used in combination [32].

## 4. Prognostic Biomarkers

Although tumor stage is an important predictor of patient outcome, survival of patients diagnosed with stage I lung cancer can also be very low [1]. Thus, there is an urgent need to understand the molecular alterations that confer a poor prognosis and to use this information to identify the high-risk patients to improve their management. 

By combining proteomics data (obtained by 2DE, MS, immunohistochemistry, and tissue microarray) with mRNA microarray data, Chen et al. identified 11 components of the glycolysis pathway as associated with poor survival in lung adenocarcinoma [33]. Among these candidates, phosphoglycerate kinase 1 (PGK1) was found to be strongly predictive of patient's survival independently of stage. PGK1 is controlled by oxygen tension, and its increased expression might reflect faster growing and more hypoxic tumors. Although PGK1 seemed to be a promising prognostic marker, its role in lung cancer is controversial. In fact, in a more recent study, overexpression of PGK1 was found to limit tumor growth in mice subcutaneously injected with the Lewis lung carcinoma cell line (LLC-1), by promoting antitumor immunity [34]. Moreover, LLC-1 cells overexpressing PGK1 showed lower invasion ability and a reduced angiogenesis induction. Therefore, the role of PGK1 in lung cancer warrants further investigations.

Since over 90% of deaths from lung cancer are attributable to metastases [35], key proteins involved in this process could represent important prognostic markers. It has been reported that upregulation of annexin A3 (ANXA3), a member of a family of calcium- and phospholipid-binding proteins, which has been related to cancer metastasis via promoting angiogenesis, was significantly associated with advanced clinical stage, lymph node metastasis, increased relapse rate, and decreased overall survival in lung adenocarcinoma, as demonstrated by 2D-DIGE, MS, Western blotting, and immunohistochemistry [36]. Thus, given its important role in lung adenocarcinoma progression, ANXA3 might serve as a novel prognostic biomarker for this cancer.

The metastatic phenotype of lung cancer seems to be also related to altered levels of S100A11, a member of S100 family of proteins, which are small calcium-binding proteins that have been implicated in prognosis and risk of metastasis in several tumor types [37]. Comparative proteomics analysis of two NSCLC cell lines, the nonmetastatic CL1-0 and highly metastatic CL1-5, performed by 2DE followed by matrix-assisted laser desorption ionization-time of flight (MALDI-TOF)/MS and MS/MS and validated by RT-PCR and Western blotting, revealed an upregulation of S100A11 in metastatic CL1-5 cells [38]. Moreover, immunohistochemical analyses in NSCLC tissues showed that upregulation of S100A11 was significantly associated with higher TNM stage and positive lymph node status, indicating that S100A11 might be an important regulatory molecule in promoting invasion and metastasis of NSCLC.

Altered expression of S100A6, another member of the S100 family, seems to be also implicated in NSCLC progression [39]. In particular, elevated levels of this protein, evaluated by surface-enhanced laser desorption ionization- (SELDI-) MS in tumor cell lysates, plasma, and pleural effusions and by immunohistochemistry on tissue microarrays, showed a trend of longer survival compared with S100A6-negative cases. Thus, although S100A6 and S100A11 belong to the same family of proteins, they have opposite roles in lung cancer progression. Indeed, S100A6 has been proposed to have a proapoptotic function [40]. 

Cytoskeletal reorganization is a central process regulating cell movement and metastasis, and therefore a number of cytoskeletal proteins have been proposed as potential cancer prognostic markers. For instance, the increased expression of cytokeratins (CKs), a family of cytoskeletal intermediate filaments, has been suggested to play a role in carcinogenesis, by promoting cellular architecture reorganization during tumor development and progression [41]. A number of isoforms of CK 7, 8, 18, and 19 were found at higher levels in adenocarcinoma samples compared to uninvolved adjacent tissues, by 2DE and MS analysis. Interestingly, specific isoforms of the four types of CK were associated with unfavorable prognosis. In a more recent study, CK18 plasma level has been compared in patients with NSCLC and benign lung diseases and healthy donors by ELISA assays, in order to explore the potential diagnostic and prognostic role of this CK in comparison with a fragment of cytokeratin-19 (CYFRA21-1), a well-established diagnostic marker for lung cancer [42]. Although CYFRA21-1 was a more accurate diagnostic marker, survival analyses showed that CK18 was a stronger prognostic factor. 

Other cytoskeletal proteins found to be correlated with a poor prognosis in lung cancer are nonmuscle myosin IIA, a major component of the actomyosin cytoskeleton contributing to cell contraction during migration, and vimentin, an intermediate filament protein involved in epithelial-mesenchymal transition, which is a process at the basis of invasive and metastatic behavior [43]. 

Phosphohistidine phosphatase (PHP14) was proposed to be another lung cancer prognostic marker, regulating cell migration and invasion by cytoskeleton rearrangement [44]. Indeed, it has been shown that PHP14 knockdown in highly metastatic lung cancer cells (CL1-5) inhibited migration and invasion, whereas its overexpression in NCI H1299 cells induced these processes. Moreover, comparative proteomics experiments, conducted in PHP14-knockdown and control CL1-5 cells, revealed changes in the expression of several proteins involved in actin cytoskeletal reorganization, thus suggesting that PHP14 prometastatic role is mediated by a cytoskeleton rearrangement. 

Finally, other potential prognostic markers involved in cytoskeleton regulation are calmodulin, a protein suggested to be implicated in cytoskeletal alterations during cell death, thymosin *β*4, a regulator of actin polymerization whose overexpression seems to stimulate lung tumor metastasis, thymosin *β*10, and cofilin-1, two regulators of actin dynamics [45]. These proteins, tested in combination, proved to be useful to predict NSCLC patients' outcome. 

## 5. Biomarkers for Treatment Response

Chemotherapy is the standard of care for most lung cancer patients. Since only a minority of patients benefit from any particular chemotherapy treatment, the identification of molecular markers predicting positive or negative clinical outcome upon chemotherapy treatment is needed. 

In a recent study, a serum peptidome profiling, performed by MALDI-TOF-MS in patients treated with cisplatin-gemcitabine in combination with the proteasome inhibitor bortezomib, revealed a 13-peptide signature distinguishing, with high accuracy, sensitivity, and specificity, patients with short versus long progression-free survival (PFS) [46]. Moreover, a 5-peptide signature could separate patients with a partial response versus nonresponders. Long duration of PFS was strongly associated with tumor response to treatment, suggesting that the survival signature is predictive of therapy outcome rather than prognostic. It has been hypothesized that the differentially expressed peptides are generated from common serum proteins following cleavage by specific exopeptidases, whose different activities contribute to generate cancer type-specific serum peptides [47]. Thus, blood proteins are the source of surrogate biomarkers because these proteins are only substrates for the real biomarkers, that is, proteases.

The epidermal growth factor receptor (EGFR) tyrosine kinase is an important target for treatment of NSCLC, and EGFR-inhibitor-based therapies showed promising results [48, 49]. In particular, gefitinib and erlotinib are selective inhibitors of the EGFR currently used in NSCLC treatment. However, only a subfraction of patients respond to EGFR inhibitors, although most NSCLC cases express EGFR. Therefore, several studies have been focused on the identification of protein signatures to select candidate patients, who are likely to benefit from treatment with these inhibitors [50–52]. In the study conducted by Taguchi et al. [50], serum analysis, performed using MALDI-MS in NSCLC patients treated with gefitinib and erlotinib, revealed an 8-peak profile as predictive of outcome. This 8-peak signature has been commercially launched and its clinical relevance is being validated in a phase III clinical trial [2].

## 6. Conclusions

Although proteomics methods are improving rapidly and the development of high-throughput platforms gave promising results, at present a comprehensive proteomics signature is still not achievable. In fact, cancer protein profiling can be very complex due to the highly variable protein concentrations, which render difficult the detection of low-abundance tumor proteins, and the extreme biochemical diversity of proteins, with a number of different protein forms that exceed several hundred thousand. In most proteomics studies the number of detectable signals is around 1000–3000, and therefore it is clear that future technological innovations are needed to better profile cancer cells by measuring a significant fraction of the proteome [8]. A number of studies have been devoted to improve lung cancer biomarker discovery by providing new technologies. For instance, Toyama and colleagues demonstrated that serum protein deglycosylation improves the quantitative performance of shotgun proteomics [53]. Moreover, in a recent study a multivariate calculation method has been suggested as a tool to differentiate lung cancer tissues, even in an early stage, and control tissues [54]. A further powerful methodology in the search for novel disease biomarkers has been proposed to be the activity-based proteomics [55].

Despite the technical difficulties, the list of candidate biomarkers for lung cancer is rapidly growing. However, there is a great need to interpret information from this data complexity to generate biologically relevant hypotheses. In fact, for many of the identified proteins the functional role in lung tumorigenesis is not yet known and a solid clinical validation is still lacking. Nevertheless, it is likely that some of these candidate biomarkers will serve to identify new possible therapeutic strategies.
